# Systematic review of the radiomics quality score applications: an EuSoMII Radiomics Auditing Group Initiative

**DOI:** 10.1007/s00330-022-09187-3

**Published:** 2022-10-25

**Authors:** Gaia Spadarella, Arnaldo Stanzione, Tugba Akinci D’Antonoli, Anna Andreychenko, Salvatore Claudio Fanni, Lorenzo Ugga, Elmar Kotter, Renato Cuocolo

**Affiliations:** 1 Department of Advanced Biomedical Sciences, University of Naples “Federico II”, Naples, Italy; 2 Institute of Radiology and Nuclear Medicine, Cantonal Hospital Baselland, Liestal, Switzerland; 3 Research and Practical Clinical Center for Diagnostics and Telemedicine Technologies of the Moscow Healthcare Department, Moscow, Russia; 4 Department of Translational Research, Academic Radiology, University of Pisa, Pisa, Italy; 5 Department of Radiology, Medical Center - University of Freiburg, Faculty of Medicine, University of Freiburg, Freiburg, Germany; 6 Department of Medicine, Surgery, and Dentistry, University of Salerno, Baronissi, Italy; 7 Augmented Reality for Health Monitoring Laboratory (ARHeMLab), Department of Electrical Engineering and Information Technology, University of Naples “Federico II”, Naples, Italy

**Keywords:** Systematic review, Diagnostic imaging, Big data, Radiomics, Radiomics quality score

## Abstract

**Objective:**

The main aim of the present systematic review was a comprehensive overview of the Radiomics Quality Score (RQS)–based systematic reviews to highlight common issues and challenges of radiomics research application and evaluate the relationship between RQS and review features.

**Methods:**

The literature search was performed on multiple medical literature archives according to PRISMA guidelines for systematic reviews that reported radiomic quality assessment through the RQS. Reported scores were converted to a 0–100% scale. The Mann-Whitney and Kruskal-Wallis tests were used to compare RQS scores and review features.

**Results:**

The literature research yielded 345 articles, from which 44 systematic reviews were finally included in the analysis. Overall, the median of RQS was 21.00% (IQR = 11.50). No significant differences of RQS were observed in subgroup analyses according to targets (oncological/not oncological target, neuroradiology/body imaging focus and one imaging technique/more than one imaging technique, characterization/prognosis/detection/other).

**Conclusions:**

Our review did not reveal a significant difference of quality of radiomic articles reported in systematic reviews, divided in different subgroups. Furthermore, low overall methodological quality of radiomics research was found independent of specific application domains. While the RQS can serve as a reference tool to improve future study designs, future research should also be aimed at improving its reliability and developing new tools to meet an ever-evolving research space.

**Key Points:**

*• Radiomics is a promising high-throughput method that may generate novel imaging biomarkers to improve clinical decision-making process, but it is an inherently complex analysis and often lacks reproducibility and generalizability.*

*• The Radiomics Quality Score serves a necessary role as the de facto reference tool for assessing radiomics studies.*

*• External auditing of radiomics studies, in addition to the standard peer-review process, is valuable to highlight common limitations and provide insights to improve future study designs and practical applicability of the radiomics models.*

**Supplementary Information:**

The online version contains supplementary material available at 10.1007/s00330-022-09187-3.

## Introduction

The overwhelming enthusiasm toward radiomics is emphasized by the ever-growing number of publications in the field [1, 2]. This high-throughput strategy to mine quantitative data from medical images searching for novel biomarkers and to generate decision-support models is deemed a feasible approach to overcome the limitations of conventional image interpretation, particularly in oncology [3–5]. The potential applications of radiomics are seemingly endless across all imaging modalities, and according to a survey study, the future physicians are confident that advanced computer-aided image analyses will revolutionize radiology for the best [6–9].

Nevertheless, after nearly a decade of research, translation of radiomics into clinical practice remains a distant prospect, and there are many unanswered questions about the potential availability of commercial radiomics tools [10]. Additionally, reasonable concerns have also been raised that we might be overlooking negative, unpublished, but potentially valuable results, i.e., publication bias [11].

Radiomics is a complex multi-step process, and within each step there are methodological challenges to overcome in order to ensure the robustness of model’s findings, while reproducibility and generalizability are often compromised [12–14]. Aiming to untangle this methodological complexity and streamline the structure of radiomics pipelines, a set of recommendations was released in 2017 along with a proposal of a “quality seal” for published results named Radiomics Quality Score (RQS) [15]. Although there is still room for improvement, the RQS has been embraced by the scientific community and has been mainly used to assess the methodological quality of previously published radiomics studies in the setting of systematic reviews [16].

The RQS consists of 16 items, with a total score ranging from − 8 to + 36 points. The percentage score is derived from the absolute score and obtained by dividing the total score by 36 [17]. The RQS items may also be grouped into six domains [18]. Domain 1 covers protocol quality and reproducibility in image and segmentation (items 1–4), domain 2 reporting of feature reduction and validation (items 5 and 12), domain 3 biological/clinical validation and utility (items 6, 7, 13, and 14), domain 4 performance index (items 8, 9, and 10), domain 5 demonstration of a higher level of evidence (items 11–15), and domain 6 open science (item 16).

In the present work, we aim to provide a comprehensive overview of RQS-based systematic reviews to highlight common issues and unique challenges in the vast array of radiomics applications.

## Methods

The study was registered on the International Prospective Register of Systematic Reviews database with the registration number CRD42021292310.

### Article search strategy

**Fig. 1:**
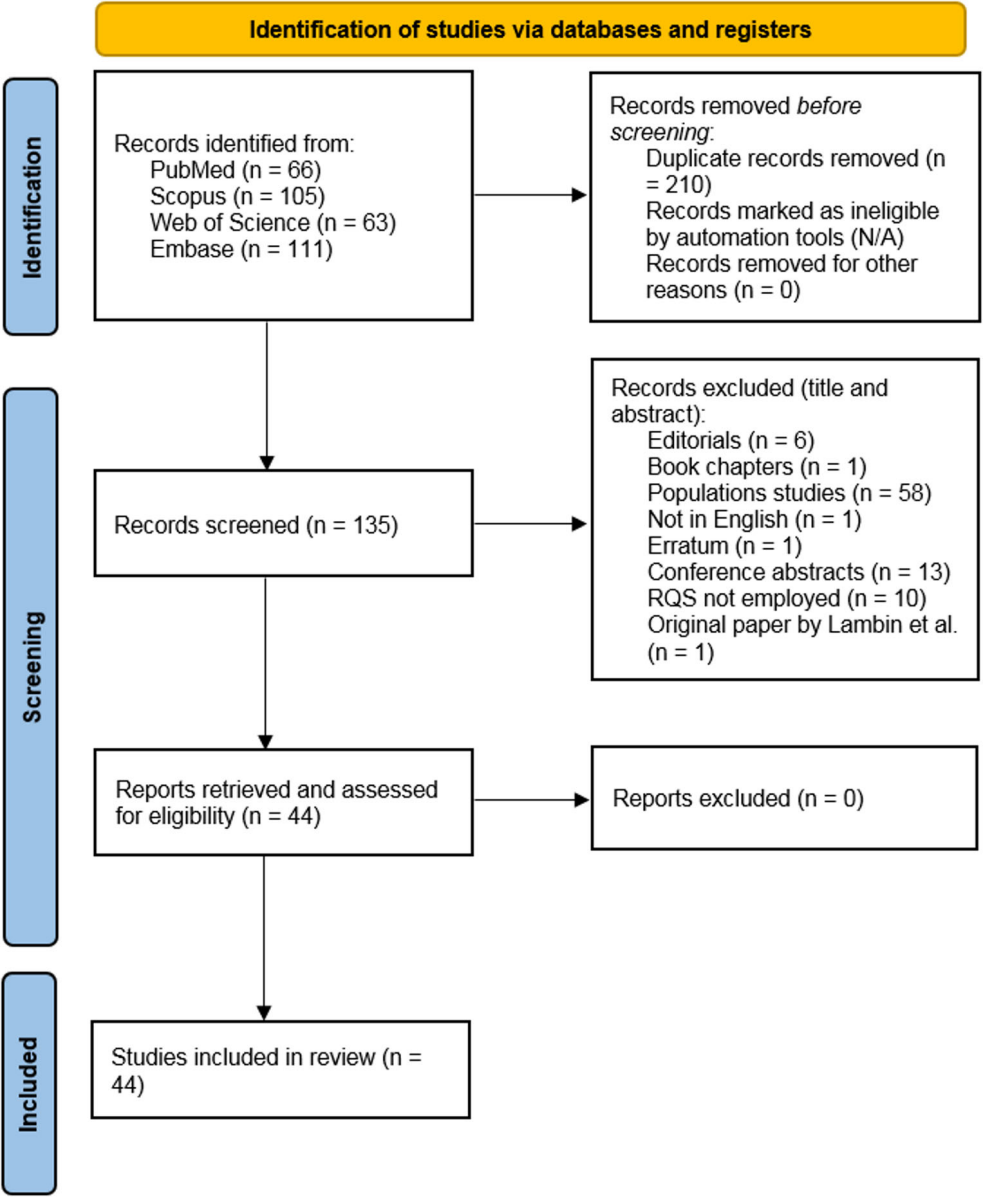
The literature research flow diagram. *Adapted from:* The PRISMA 2020 statement: an updated guideline for reporting systematic reviews [19]

The literature search was performed according to PRISMA (Preferred Reporting Items for Systematic reviews and Meta-Analyses) guidelines in the electronic databases (PubMed, Web of Science, Embase, and Scopus) using the following search query: ((“radiomics” OR “radiomic”) AND “quality” AND “score”). The systematic reviews that reported radiomic quality assessment performed according to the RQS and published until December 31, 2021, were included. Letters, editorials, duplicates, original articles, literature reviews, and RQS systematic reviews published in languages other than English were excluded from the analysis. The included articles were selected by consensus of four radiologists experienced in radiomics/texture analysis, systematic literature review, and RQS assessment. In Fig. 1, the results of the article selection are shown.

### Data extraction and analysis

The RQS comprises six domains (image protocol, radiomics features extraction, data analysis and statistics, model validation, clinical validity, and open science) and 16 items. By assessing each item, a final score will be determined, which is presented on a scale of − 8 to 36 and can be converted to a percentage (where scores below 0 accepted as 0 and 36 equals 100%), as reported by Lambin et al [15]. Details of the RQS domains and items along with the scores can be found in the supplementary materials. The same group of radiologists (A.S. and L.U.: 5 years of experience, C.F. and T.A.D.: 2 years each) also extracted the data from all included studies and collected the median or mean of RQS from included studies.

Moreover, the included studies were classified based on the following characteristics: (1) oncological versus non-oncological target; (2) neuroradiology versus body imaging focus; (3) single versus multiple imaging modalities; (4) aim of the studies that included to systematic reviews (characterization, detection, prognosis prediction, or other).

### Statistical analysis

All the analyses were performed using the mean RQS percentage scores reported in each systematic review, after conversion of the median values to corresponding means [20]. When necessary, raw data from included studies were retrieved to calculate mean RQS percentage scores. The relation between the study quality and article subgroups was tested. The normality of the data distribution was assessed with the Kolmogorov-Smirnov test. To compare variables with a non-normal distribution, a Mann-Whitney test was performed. The Kruskal-Wallis test was used to compare multiple continuous variables. Continuous variables are presented as median and interquartile range (IQR), categorical ones as count and percentage. All statistical analyses were performed using SPSS (SPSS version 27; SPSS). Alpha level was set to 0.05.

## Results

### Literature review

The initial literature research resulted in 345 articles, of which 210 were duplicates. Finally, 44 studies were selected from the remaining 135 because 91 articles did not meet the inclusion criteria. The study flowchart is shown in Fig. 1 and all systematic reviews included in this study are listed in Table 1.

**Table 1:** Characteristics of the included systematic reviews

First author	Year	Journal	Organ system	*n* of studies	Mean %
Abdurixiti [21]	2021	British Journal of Radiology	Lung	6	35
Abunahel [22]	2020	European Radiology	Pancreas	72	29
Bhandari [23]	2020	Abdominal Radiology	Kidney	13	31
Bhandari [24]	2020	American Journal of Neuroradiology	Brain	14	29
Calabrese [25]	2021	Journal of Cancer Research and Clinical Oncology	Breast	10	30
Carbonara [26]	2021	Journal of Oncology	Head and Neck	8	21
Castillo [27]	2020	Cancers	Prostate	13	51
Chen [28]	2021	European Journal of Nuclear Medicine and Molecular Imaging	Lung	10	30
Chetan [29]	2020	European Radiology	Lung	14	21
Crombe [30]	2020	European Journal of Radiology	Soft tissue	52	18
Davey [31]	2021	European Journal of Radiology	Breast	41	18
Fornacon-wood [32]	2020	Lung Cancer	Lung	43	21
Granzier [33]	2019	European Journal of Radiology	Breast	16	12
Harding-theobald [34]	2021	Alimentary Pharmacology and Therapeutics	Liver	54	25
Janssen [35]	2021	Annals of Surgery	Pancreas	23	21
Kao [36]	2021	In Vivo	Esophagus	7	26
Kao [37]	2021	Diagnostics	Lung	7	39
Kendrick [38]	2021	Frontiers in Oncology	Prostate	17	23
Kim [39]	2021	Neuro-Oncology Advances	Brain	7	3
Kozikowskim [40]	2021	European Urology Focus	Bladder	8	41
Lecointre [41]	2021	European Journal of Surgical Oncology	Uterus	17	15
Muhlbauer [42]	2021	Cancers	Kidney	113	14
Nardone [43]	2021	Radiologia Medica	Multiorgan	48	21
Park [44]	2020	European Radiology	Multiorgan	77	26
Park [18]	2020	BMC Cancer	Brain	51	22
Ponsiglione [45]	2021	European Radiology	Cardiovascular	53	12
Sanduleanu [17]	2018	Radiotherapy and Oncology	Multiorgan	41	22
Shi [46]	2021	European Journal of Radiology	Lung	28	19
Spadarella [47]	2021	European Journal of Radiology	Pharynx	24	21
Staal [48]	2021	Clinical Colorectal Cancer	Large bowel	76	13
Stanzione [49]	2020	European Journal of Radiology	Prostate	73	23
Tabatabaei [50]	2021	Oncology	Brain	18	76
Ugga [51]	2021	Neuroradiology	Brain	23	19
Ursprung [52]	2020	European Radiology	Kidney	57	9
Valdora [53]	2018	Breast Cancer Research and Treatment	Breast	17	33
Wakabayashi [54]	2019	Hepatology International	Liver	23	23
Walls [55]	2021	Clinical Oncology	Lung	44	17
Wang [56]	2020	European Radiology	Hematology	45	14
Wang [57]	2021	Cancers	Liver	22	28
Wesdorp [58]	2020	European Journal of Nuclear Medicine and Molecular Imaging	Gastrointestinal	60	23
Wesdorp [59]	2021	Surgical Oncology	Gastrointestinal	14	19
Won [60]	2021	European Journal of Radiology	Brain	25	15
Won [61]	2020	Korean Journal of Radiology	Brain	26	10
Zhong [62]	2021	European Radiology	Bone	12	20

### Study features and subgroup analysis

**Fig. 2:**
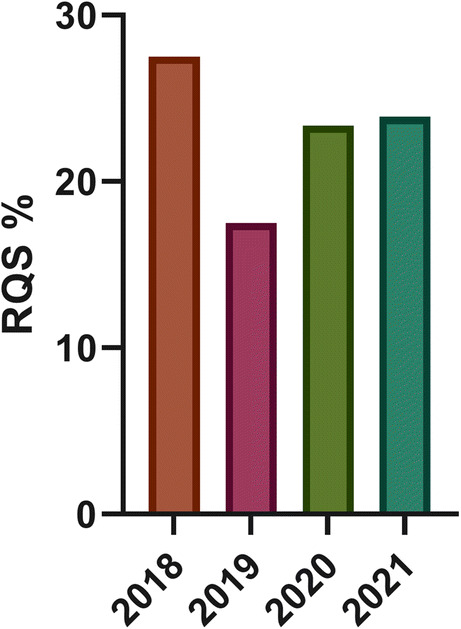
Bar plot reporting the mean RQS percentage score by year of publication

**Fig. 3:**
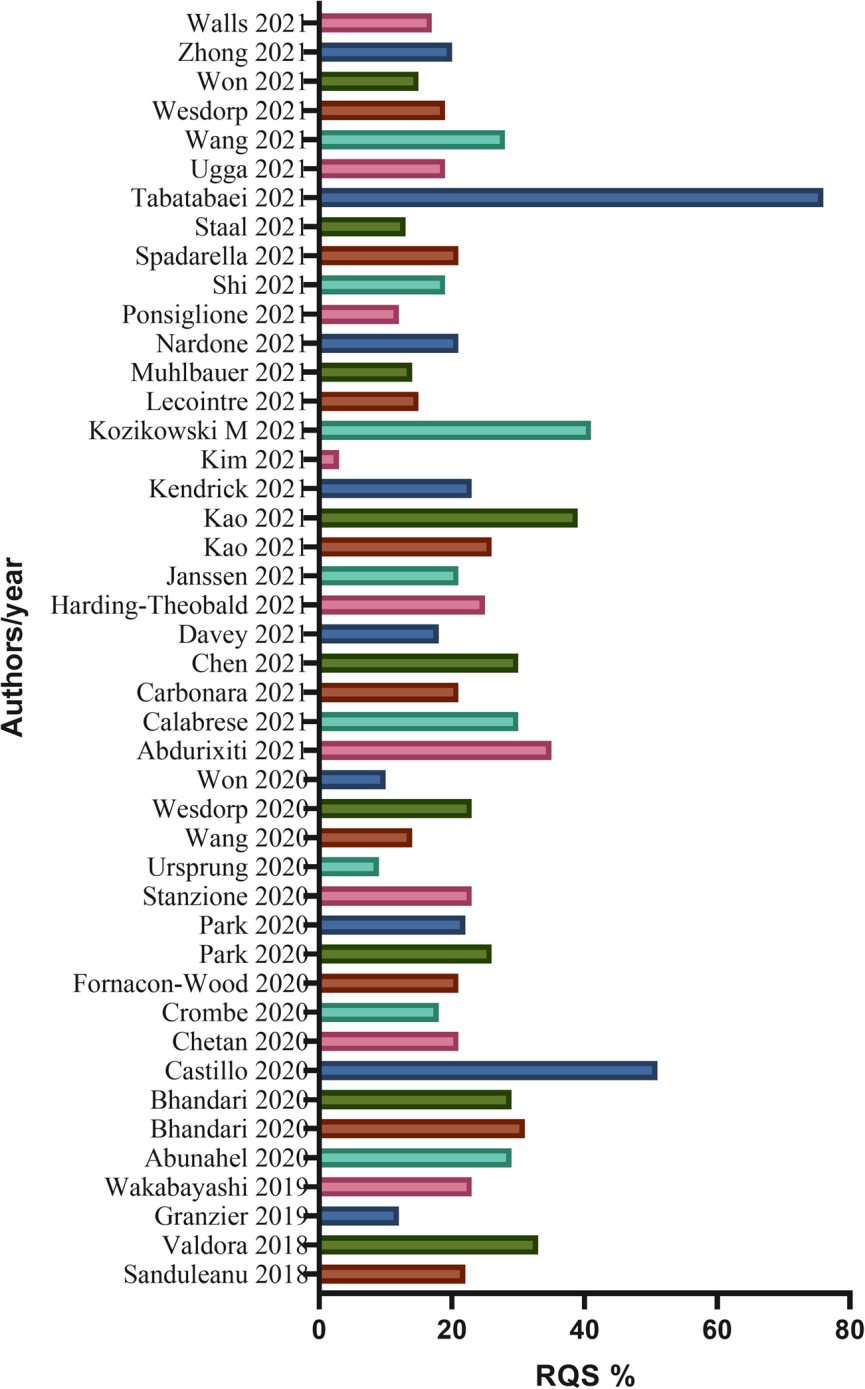
Mean of RQS percentage score of each review included in this study

**Fig. 4:**
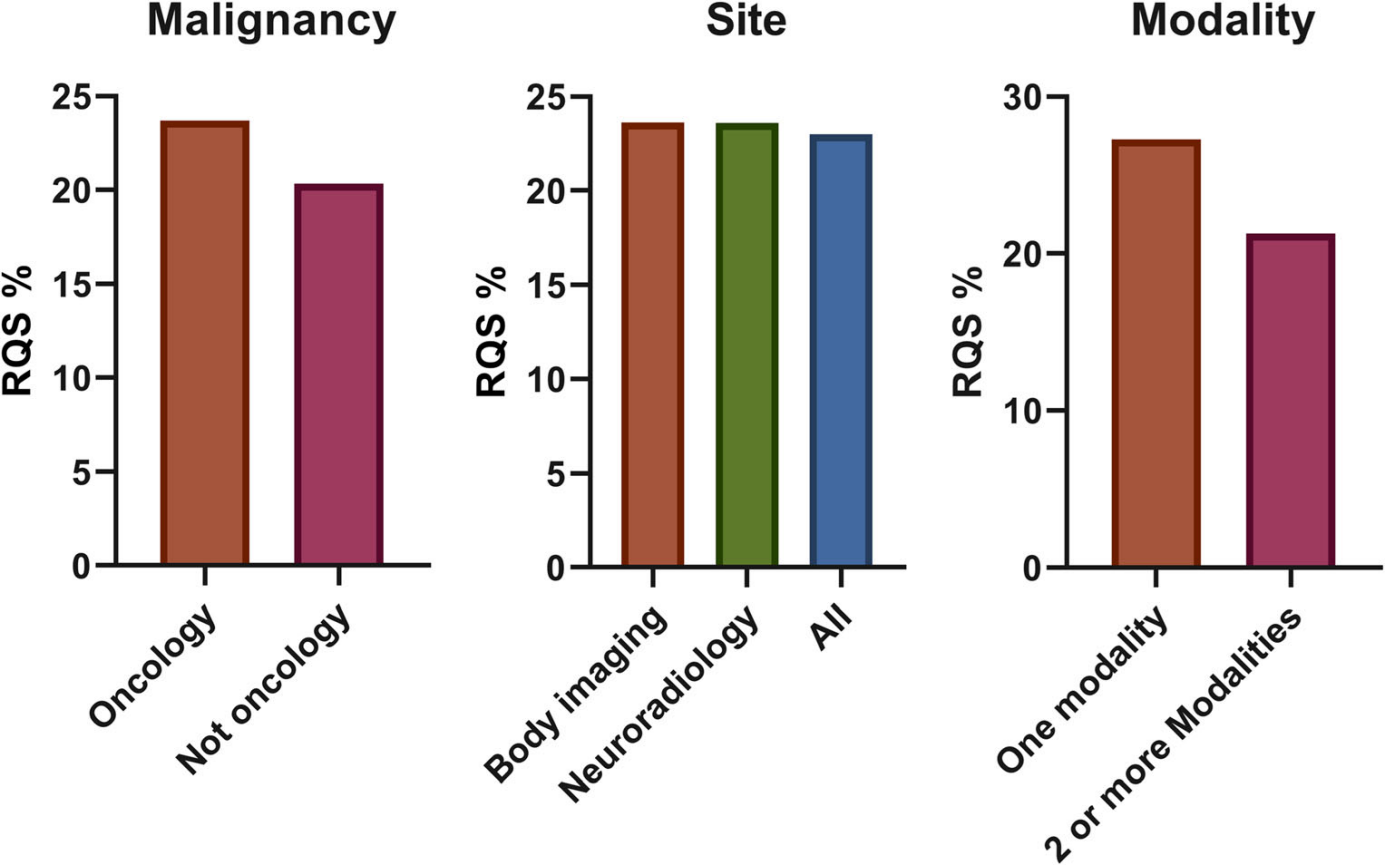
RQS percentage score for different review subgroups

**Fig. 5:**
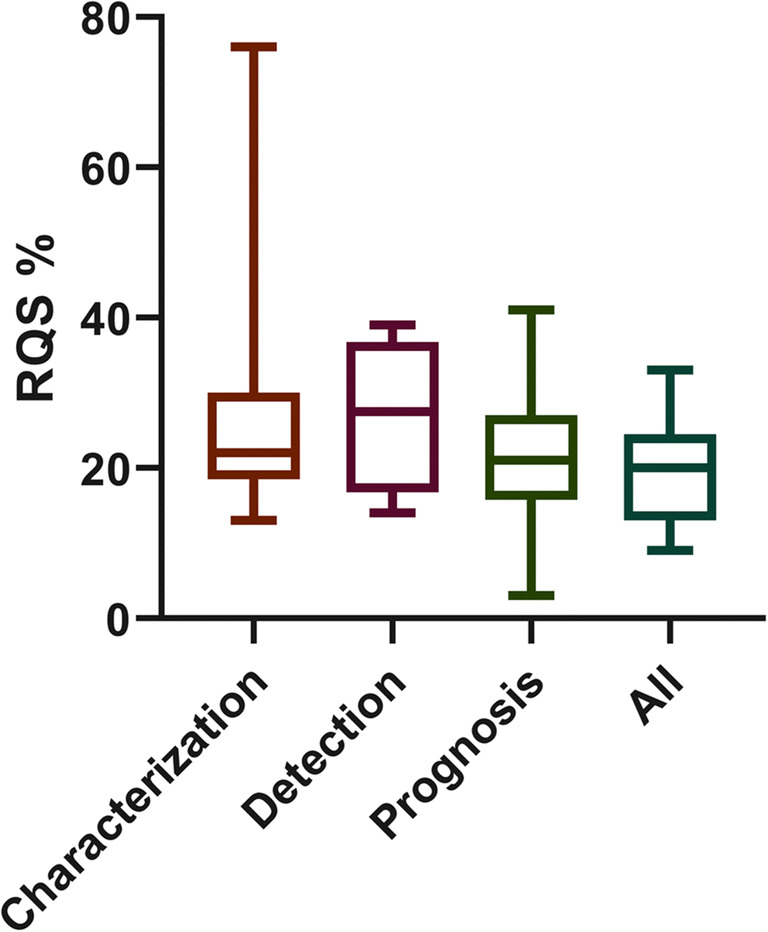
Box plot depicting the distribution of RQS percentage score by aim of the studies included in the systematic reviews

Study features are summarized in Table 2. Additional details are reported in the supplementary materials. The median of RQS was 21.00% (IQR = 11.50). In 36 systematic reviews, quality assessment was performed by 2 or more readers (36/44, 81%). Discrepancies were evaluated in different ways: 11/44 studies assessed agreement intraclass correlation coefficient (ICC) or Cohen’s kappa, and 2 authors reported the mean of RQS score, while 23 authors chose consensus for reproducibility evaluation. The remaining studies (8/44, 18%) did not specify the reproducibility test. As shown in Fig. 2, the highest mean RQS score of 27.50% reported in systematic reviews published in the year 2018 while the lowest RQS was reported in 2019. Most of the review articles focused on oncological radiomics studies (40/44, 90%); ten out of forty-four (22.7%) reviews were focused on neuroradiology radiomics articles. Twenty-five percent of systematic reviews included 50 or more studies in the main analysis (11/44), with a range between 6 and 113 articles included. Furthermore, the systematic reviews with a body imaging topic included 33 articles on average, while neuro-imaging reviews covered a mean of 20 studies. Notably, 38% (17/44) of articles were focused on one imaging technique, in which most of them selected MRI (16/44, 36%). In Fig. 2, mean RQS% of selected systematic reviews in each year were reported, while in Fig. 3, the mean RQS% of each review included are described. The mean RQS% separated according to the systematic review characteristics is shown in Figs. 4 and 5.

**Table 2:** Median RQS percentage scores for review subgroups

Characteristics	Statistical analysis	Median RQS% (*n* of studies)	*p* value
Body/neuroradiology/other*	Anova Kruskal-Wallis	23.6 (31)/23.6 (10)/23.0 (3)	0.586
Oncology/not oncology	Mann-Whitney	27.3 (40)/20.3 (4)	0.396
Single modality/2 or more modalities	Mann-Whitney	27.3 (17)/20.3 (27)	0.277
Characterization/detection/prognosis/other**	Anova Kruskal-Wallis	27.2 (14)/27.0 (4)/21.3 (10)/19.6 (13)	0.413

*Systematic reviews not covered by neuroradiology or body imaging categories

**Systematic reviews not covered by characterization/detection/prognosis target

The results of the subgroup analysis according to the systematic review features did not demonstrate any significant difference between subgroups (Figs. 4 and 5).

## Discussion

In recent years, the number of published radiomics studies has been increasing exponentially, notably in the field of oncological imaging [63]. This is mainly due to the promising results in this area, made possible thanks to the use of artificial intelligence/machine learning approaches instead of classical statistical tests and expert systems, capable of analyzing such a large amount of quantitative data and producing classification or prediction models. As a result of the overwhelming number of studies in this field, the need for providing research guidelines has arisen to ensure better standardization and homogenization. In this context, the image biomarker standardization initiative (IBSI), an independent international collaboration, has been working toward standardizing the extraction of image biomarkers from acquired imaging. IBSI provides an image biomarker nomenclature and specific feature definitions, as well as a general image processing workflow, tools for verifying radiomics software implementations, and reporting guidelines for radiomics studies [64]. Together with the need for standardization, the need for a tool for qualitative assessment and comparison of extremely heterogeneous radiomics methodologies has also arisen. In relation to this question, Lambin et al introduced the radiomics quality score (RQS) in 2017 [15]. The RQS followed previous efforts that did not focus on radiomics, such as the transparent reporting of a multivariable prediction model for individual prognosis or diagnosis statement published in 2015 [65]. The aim of the RQS is to evaluate the methodological quality of radiomics-based investigations, identifying high-quality results as well as issues limiting their value and applicability. However, as stated by the creators of the RQS themselves, this score was not conceived as an external auditing tool to express a qualitative appraisal in absolute terms or to conduct systematic reviews, but rather as a practical checklist to guide researchers in study designing and to give them the possibility to justify any methodology noncompliance [16]. However, in practice, this tool has become the de facto standard for systematic reviews of the literature focused on radiomics quality assessment as confirmed by our findings. In any case, it should be acknowledged that some alternatives have been proposed, even though their use is usually sporadic [66, 67]. Additionally, several checklists have been presented in the recent literature, including the Checklist for Artificial Intelligence in Medical Imaging, Minimum Information for Medical AI Reporting checklist, and currently under development artificial intelligence extensions of the Transparent Reporting of a multivariable prediction model of Individual Prognosis Or Diagnosis statement and the Prediction model Risk Of Bias Assessment Tool [68–70]. However, these are not tailored for use in radiomics specifically, but are focused on machine learning modeling and correct management of the data in relation to model bias. Also, their nature as checklists does not allow a formal methodological quality score, but rather an unweighted assessment of overall adherence to the included items.

Regarding the use of RQS to perform an external assessment of methodological quality in radiomics studies, its potential lack of reproducibility may represent an issue. Only in a minority of the studies included in our systematic review, the authors performed an assessment of the RQS’s inter-reader reproducibility, either through the intraclass correlation coefficient or Cohen’s K. In several cases, a consensus approach was employed with multiple raters, which may represent a valid solution to ensure the score’s reliability. The assessment of the RQS’s reproducibility, also accounting for differences in raters’ experience levels, may represent an avenue of future research of itself if its use for systematic study quality auditing will continue. It would also be ideal to identify a standard practice on this topic, either requiring inclusion of an inter-reader reproducibility analysis or a consensus approach for all future RQS-based reviews. This would mitigate concerns regarding possible biases in the final scores.

Another limitation of the RQS pertains to its use for deep learning–based studies. Several authors have used the RQS to assess the quality of this type of research, but the RQS items are not perfectly suited for this task. On one hand, it can be argued that computer vision neural networks, especially when based on convolutions, essentially extract quantitative features that can be assimilated to typical radiomics parameters. However, the processing of this data diverges from the classical feature processing, selection, and model tuning pipeline of radiomics. Probably, the appropriateness of the RQS should be evaluated on a case-by-case basis for deep learning research. In the future, it could be appropriate to develop dedicated tools tailored to address both classical machine learning and deep learning radiomics analyses, sharing part of the items but diverging as necessary to avoid biases [41]. In this setting, the information contained in the previously mentioned healthcare artificial intelligence modeling checklists could prove valuable to complement the original RQS.

It should also be noted that the average RQS across all included reviews was low (median = 21.00%; IQR = 11.50). This, not only, supports the conclusions drawn by each individual review that the methodological quality and/or thoroughness of its presentation within scientific studies is still far from ideal but also raises questions about the appropriateness of RQS as a qualitative quality measure of radiomics research. The former is also supported by a recent investigation of methodological issues in machine learning research across different domains, including medicine and radiology, which also supports that inappropriate use of data analysis techniques indeed constitutes a critical issue [71], while the latter stems from the low variance of reported RQSs. This limitation of the current landscape of radiomics research represents undoubtedly one of the main factors preventing the translation of these tools to clinical decision support systems. Furthermore, as awareness of this problem grows throughout the medical imaging community, skepticism in the general public will only increase. This negative perception will probably persist for some time even if the quality and reliability of radiomics research improve in the near future. Researchers active in this field should therefore be particularly incentivized in improving the presentation and clarity of their methods and ease the reproduction of their experiments to foster a more positive environment and facilitate rather than hinder the adoption of radiomics-based software in clinical practice. As shown in our review, unfortunately, this does not seem to be currently the case. In this setting, journals and reviewers will probably need to take a more active role in raising the bar for minimal quality of radiomics research to be published. Guiding researchers toward a greater focus on investigations aiming at improved clinical outcomes rather than technical feasibility alone would also be a positive development. Some editors and journals have already begun to move in this direction, and it is desirable for this trend to spread at least to the more visible publications in our field [68, 72, 73].

Based on the results reported in the RQS systematic reviews included in this investigation, some common trends emerge. Some points were lacking in all or almost all instances, such as cost-effectiveness and decision curve analyses. Prospectively designed studies are also very rare, which is a common situation across radiology research compared to other clinical specialties. More worrisome, there is still a relevant number of studies that do not perform a validation of a final model, without retraining (e.g., as done in cross-validation). While cross-validation is a valuable tool to extract more information from smaller datasets and provide a better estimate of general performance of a pipeline, it is also true that it does not provide a univocal assessment of a model’s deployment in a real-world setting. The pairing of cross-validation for model development and pipeline tuning and external validation of a definite model on a diverse dataset is probably the best solution. However, understandably, dataset size has to be adequate to allow both the training and external validation data to appropriately represent the model’s general population target. It should also be noted that the RQS also addresses some items only superficially, such as feature reduction. It does not include an assessment of the appropriateness of the techniques applied or the resulting dataset’s size in comparison to the number of instances available for training. This could lead to an overestimation of the study’s RQS score, as feature reduction accounts for either a − 3 or + 3 score out of the maximum of 36. Finally, we wish to highlight the lack of openness in many of the radiomics studies. Sharing the models and, ideally, the data used to train them is essential to allow correct assessment of their validity and validation on data from institutions different from those where they were developed. These steps are essential to grow trust in radiomics research and allow development of clinical decision support tools integrating these types of models.

Our systematic review presents some limitations that should be acknowledged. We did not aggregate the singular item data from each RQS-based review included in our study. This was partly due to the significant divergence in methods used to perform the rating (consensus, single reader). Also, it was not our intention to substitute the original studies in their topic-specific assessment, but rather to provide a wider overview of the current radiomics research state of the art. Therefore, we chose to aggregate the overall RQS percentage scores to obtain this result.

In conclusion, our review confirms the common sentiment that radiomics research quality must be increased in the near future as it is currently unsatisfactory independently of the study topic. External auditing of these investigations, in addition to the standard peer-review process, is valuable to highlight common limitations and provide insights to improve future study designs. The RQS serves a necessary role as the de facto reference tool for this task, but future research should be aimed at improving its reliability and developing new tools to meet an ever-evolving research space.

## Supplementary information

ESM 1 (DOCX 24 kb)

ESM 2 (XLSX 12 kb)

## Abbreviations

IBSI: Image biomarker standardization initiative
ICC: Intraclass correlation coefficient
IQR: Interquartile range
RQS: Radiomics quality score

## Declarations

### Guarantor

The scientific guarantor of this publication is Prof. Renato Cuocolo, MD, PhD.

### Statistics and biometry

No complex statistical methods were necessary for this paper.

### Methodology

• systematic review

• performed at multiple institutions
